# Is reflux hypersensitivity truly a functional gastrointestinal disorder? A retrospective cross-sectional study

**DOI:** 10.1371/journal.pone.0316226

**Published:** 2025-01-17

**Authors:** Yanping Wu, Siyu Liao, Tianyao Qu, Jiaxuan Zhou, Fei Dai, Bin Qin, Xiaoyu Xu, Jun Zhang, Yan Cheng

**Affiliations:** Department of Gastroenterology, Second Affiliated Hospital of Xi’an Jiaotong University, Xi’an, China; Quaid-i-Azam University Islamabad: Quaid-i-Azam University, PAKISTAN

## Abstract

**Background:**

According to Rome IV, reflux hypersensitivity (RH) represents a novel form of functional esophageal disorder. This study was designed to compare the clinical features of three types of endoscopic-negative heartburn: RH, nonerosive reflux disease (NERD), and functional heartburn (FH).

**Methods:**

Patients with heartburn in a medical center from 01/01/2017 to 10/31/2021 were included. This article presented a blinded retrospective analysis of 24 h MII–pH and HRM tracings from patients with NERD, RH, and FH to compare their clinical characteristics.

**Results:**

A total of 118 patients were included in the study. There were no significant differences in age, sex, BMI, smoking status, or drinking history among RH, NERD and FH. Functional dyspepsia (FD) symptoms were more prone to exist in FH than in NERD (*P* < 0.05), whereas hiatal hernia was more prevalent in NERD and RH than in FH (*P* < 0.05). The incidence of anxiety and depression gradually increased in the NERD, RH, and FH groups (*P* >  0.05). The distal MNBI and PSPW index of the NERD and RH groups were lower than those of the FH group (*P* < 0.05). The distal MNBI showed good diagnostic potential.

**Conclusions:**

Clear pathological alterations, which are distinct from those of other FGIDs, are observable in RH. It might be inappropriate to categorize RHs within FGIDs.

## Introduction

The prevalence of gastroesophageal reflux disease (GERD), which affects all age groups and sexes, is estimated to be 8%–33% worldwide [1]. Seventy percent of patients have no esophageal mucosal injury according to endoscopy, which is collectively referred to as “endoscopically negative heartburn” [2]. Although proton pump inhibitors (PPIs) are regarded as the essential therapy for GERD, 40–50% of endoscopically negative heartburn patients have a poor response to PPI therapy [3], with an approximately 20% reduction in therapeutic benefit compared with patients with erosive esophagitis [4]. These patients are classified into three subtypes on the basis of the results of pH monitoring of esophageal impedance in Rome IV [5]: nonerosive reflux disease (NERD), abnormal exposure to esophageal acid, whether symptoms are related to reflux or not; reflux hypersensitivity (RH), where esophageal acid exposure is normal and symptoms are associated with reflux; and functional heartburn (FH), where esophageal acid exposure is normal and symptoms are not associated with reflux.

Notably, the Rome IV Committee separated RH from NERD subtypes on the basis of the Rome III diagnostic criteria and considered RH similar to FH as a functional esophageal disease [5], which means that the displacement of the subgroups of patients with an esophagus hypersensitive to acid or weak acid or both, from GERD to the world of functional disorders, dramatically reduces the number of patients with NERD. Indeed, if only patients with abnormal AET are identified as affected by GERD, approximately 40% of the current NERD population is excluded from this disease [6]. However, as studies have progressed, heterogeneity between RHs and FHs has gradually emerged. First, the classification of nonerosive reflux disease (NERD) on the basis of MII-pH findings is questionable because this technique is not perfect for GERD diagnosis and can be affected by false negative results and day-to-day variability. One-third of patients classified as having FH at 24 h MII-pH can be reclassified as having NERD or RH after a more prolonged, wireless pH recording period [7]. Second, previous studies revealed that the impairment of mucosal integrity is similar in RH and GERD patients and is absent in FH patients [8,9]. The frequency of esophagitis under the microscope in RH patients is much greater than that in FH patients [10]. IL-8 levels were significantly increased in RH patients, similar to previous findings in NERD patients, highlighting a shared inflammatory characteristic between RH and NERD [11,12]. Furthermore, RH patients often have worse esophageal clearance than FH patients [8,13–15]. If the exclusion of structural, inflammatory, and significant motor abnormalities remains fundamental to attributing typical esophageal symptoms to a functional condition, mild abnormalities in esophageal motility and cellular microstructure might prevent RH from being classified as an functional gastrointestinal disorder (FGID) [10]. Third, antireflux surgery is effective in treating RH, especially in cases of reduced LES pressure and hiatal hernia [16], but cannot benefit patients with FH [17]. Both antireflux surgery and endoscopic treatment should be avoided in patients with FH [18,19]. Psychopsychological factors also promote the occurrence of FH, as an increase in stressful activities, including loud noise and sleep deprivation, can increase the perception of esophageal symptoms [20,21]. Moreover, neuromodulators have established value in treating FH by altering neuronal function with a primary central action and a minor secondary peripheral action on esophageal pain [22]. However, a systematic review of the effects of antidepressants in patients with functional esophageal disorders revealed that there is limited evidence that antidepressants benefit RH patients because they may not constitute a homogeneous population on the basis of the Rome IV criteria [10]. It seems that simply classifying RHs as FGIDs is not absolutely appropriate. This study aims to examine the heterogeneity among NERD, RH, and FH.

## Materials and methods

### Study population

Patients with heartburn underwent upper gastrointestinal endoscopy, MII-pH (Ohmega dynamic impedance-pH monitoring system for esophagus, MMS of the Netherlands) and HRM (22-channel liquid perfusion esophageal high-resolution manometry system, MMS of the Netherlands) from 01/01/2017 to 10/31/2021 were included. Patients were required to discontinue PPIs, Potassium-Competitive Acid Blockers (P-CABs), and all motility-related medications for at least one week prior to undergoing MII-pH and HRM testing. The data were accessed for research purposes on 01/01/2022. The authors had no access to information that could identify individual participants during or after data collection.

### Main measurement

Those with a normal upper gastrointestinal endoscopy and abnormal AET ( >6%) and/or reflux episodes ( >80 times/day) were diagnosed with NERD. Patients with physiological reflux (normal endoscopy and acid exposure) and a positive correlation between symptoms and reflux were diagnosed with RH, and those with physiological reflux but a negative correlation between symptom and reflux were diagnosed with FH [23,24]. According to the Lyon Consensus, normal acid exposure is defined as AET < 4% [23]. A symptom index ≥50% and/or symptom association probability ≥ 95% were considered positive for symptom correlation, specifically for heartburn. The mean nocturnal baseline impedance (MNBI) was measured from the impedance channels of MII-pH. The proximal esophageal MNBI is defined as the mean impedance measured at channels located 17 and 15 cm above the dentate line. The distal esophageal MNBI is defined as the mean impedance measured at channels located 9 cm, 7 cm, 5 cm, and 3 cm above the dentate line [25]. A postreflux swallow-induced peristaltic wave (PSPW) was defined as a 50% decrease in impedance from the proximal to all distal impedance sites. The PSPW also meets the condition that the wave reaches the lowest distal impedance site, which should occur within 30 seconds after reflux. The PSPW index was calculated manually as the number of PSPWs divided by the number of total reflux events [26].

The clinical symptoms of patients were collected by consulting medical records. The Likert 4-point scale [27] was adopted to record the frequency and severity of symptoms (i.e., frequency: 0 =  none; 1 =  1 day per week; 2 =  2–3 days per week; 3 =  4–7 days per week. Severity: 0 =  none; 1 =  mild; 2 =  moderate; 3 =  severe). Symptom score =  symptom frequency score ×  symptom severity score. A more than 50% improvement in symptom score after treatment was considered as an effective PPI treatment.

Hiatal hernia was diagnosed on the basis of the Chicago Classification version 3.0 through HRM. EGJ morphology types II and III were considered indicative of hiatal hernia. Depression and anxiety were assessed using Hamilton Depression Scale and Anxiety Scale.

### Statistical methods

Categorical data were described as numbers, and continuous data with a nonnormal distribution are presented as medians (interquartile ranges [IQRs]). The data were compared via the Kruskal‒Wallis or χ^2^ test. Between-group comparisons were performed via the Mann‒Whitney U test with Bonferroni correction. Receiver operating characteristic (ROC) curves were obtained, and the area under the curve (AUC) and cutoff values were calculated to regroup RH and FH. *P* < 0.05 indicated statistical significance for each test. All the data were analyzed with SPSS software (version 26.0; IBM Corp, Armonk, NY, USA).

### Ethics

The Institutional Review Board of Xi’an Jiaotong University approved the study. (Approval number: 2021200).

## Results

### Demographic characteristics

**Fig 1:**
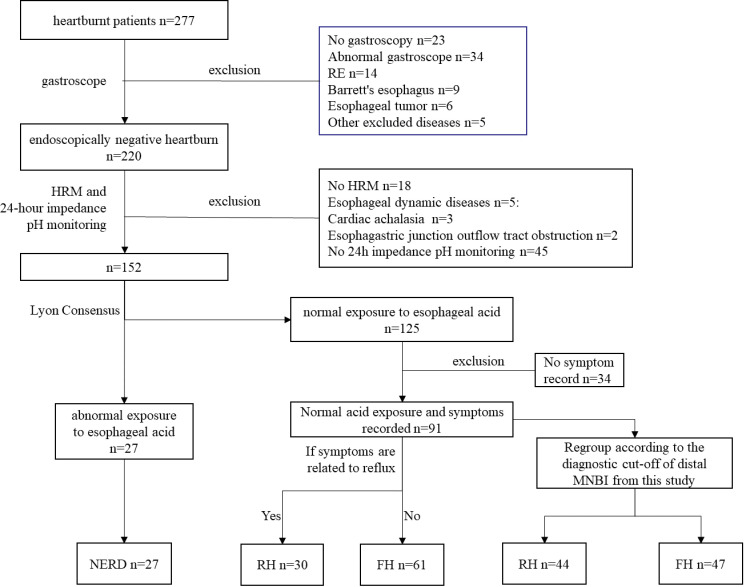
Study groups and procedures.

A total of 118 patients were ultimately included (Fig 1). There were no significant differences in age, sex, BMI, smoking, or drinking history among the NERD, RH, and FH groups (*P* > 0.05) (Table 1).

**Table 1:** Characteristic of the three groups.

Group	NERD (n = 27)	RH (n = 30)	FH (n = 61)	*P value*
Gender (male/female)	14/13	11/19	21/40	0.289
Age (year)	51.3 ± 13.8	45.5 ± 10.9	48.0 ± 12.0	0.200
BMI (kg·m^−2^)	23.4 ± 4.2	21.7 ± 2.7	21.9 ± 3.3	0.091
Smoking (Y/N)	7/20	6/24	12/49	0.790
Drinking (Y/N)	13/14	12/18	19/42	0.295
Acid regurgitation (Y/N)	22/5	22/8	43/18	0.557
Dysphagia (Y/N)	3/24	0/30^a^	1/60^a^	0.038^*^
Chest pain (Y/N)	3/24	3/27	9/52	0.783
Poststernal foreign body sensation (Y/N)	11/16	6/24	13/48	0.114
Pharyngitis (Y/N)	8/19	12/18	25/36	0.582
Chronic cough (Y/N)	7/20	5/25	11/50	0.623
Asthma (Y/N)	1/26	1/29	0/61	0.335
Globus Sensation (Y/N)	5/22	8/22	20/41	0.382

Note: ^a^*P* < 0.05 compared with NERD group. BMI: body mass index.

### Clinical symptoms

#### Esophageal symptoms.

In addition to heartburn, regulation was the most common typical esophageal symptom in all three groups, followed by retrosternal foreign body sensation, chest pain, and dysphagia. There was no significant difference in the incidence of esophageal symptoms, including regulation, chest pain, or retrosternal foreign body sensation, among the groups (*P* > 0.05). The NERD group was more prone to dysphagia than the RH and FH groups were (*P* < 0.05). None of the RH patients included in this study had dysphagia (Table 1).

#### Extraesophageal symptoms.

In all three groups, pharyngitis was the most common extraesophageal symptom. There was no significant difference in the incidence of pharyngitis or other extraesophageal symptoms among the three groups (*P* > 0.05) (Table 1).

#### FD-related symptoms.

**Fig 2:**
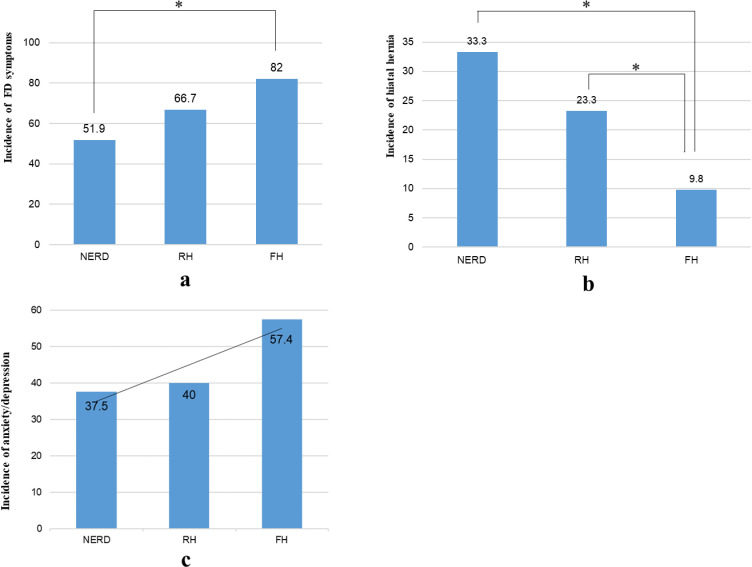
(a) Incidence rate of FD symptoms among the three groups. (b) Incidence rate of hiatal hernia among the three groups. (c) Psychological states of the three groups. * , *P*  < 0.05; ***, *P* < 0.001.

The incidence of FD symptoms, including abdominal pain, bloating after meals, belching, nausea, and vomiting, increased gradually in the NERD, RH, and FH groups. There was no significant difference between the RH and NERD groups. However, the FH group was significantly more likely to have FD symptoms than the NERD group was (*P* < 0.05) (Fig 2a).

### Hiatal hernia

Hiatal hernia was observed in all three groups, and the incidence of hiatal hernia in the NERD, RH, and FH groups also gradually decreased. Similarly, there was no significant difference between the RH and NERD groups. Compared with those in the FH group, hiatal hernias were more common in the RH and NERD groups (*P* < 0.05) (Fig 2b).

### Anxiety and depression

There was no significant difference in anxiety/depression among the three groups (*P* > 0.05). However, its incidence gradually increased in the NERD, RH, and FH groups (Fig 2c), and the prevalence of anxiety/depression was similar in the NERD and RH groups.

### Response rates to PPI treatment (placebo response not excluded)

**Fig 3:**
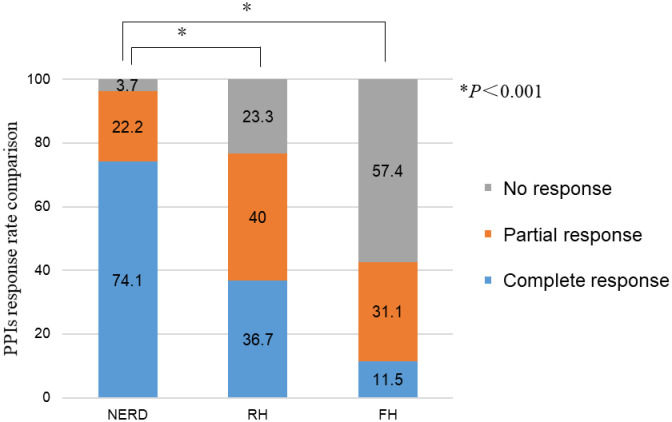
The response rates to PPIs in the three groups. No response: no relief of heartburn symptoms after 8 weeks of standard-dose PPI treatment. Partial response: partial symptom control during treatment with a standard dose of PPIs, or effective symptom control while on standard medication, with symptom recurrence within one week after discontinuation. Complete response: full resolution of heartburn symptoms after 8 weeks of standard-dose PPI treatment.

The response rates to PPIs of NERD, RH and FH were 96.3%, 76.7%, and 42.6%, respectively. Among them, 22.2% of NERD patients, 40% of RH patients, and 31.1% of FH patients experienced symptoms of recurrence after stopping PPIs. The curative effect of PPIs in NERD patients was significantly better than that in RH and FH patients (*P* < 0.001). There was no significant difference between the RH and FH groups (*P* > 0.05), but the response rates to PPIs in the FH group were much lower than those in the RH group (Fig 3).

### Parameters of HRM

The lower esophageal sphincter (LES) average resting pressure, LES residual pressure, and intact relaxation pressure in Group NERD were lower than those in Groups RH and FH (*P* < 0.05), and there were no significant differences in the remaining esophageal dynamics parameters among the three groups (*P* > 0.05) (Table 2).

**Table 2:** Parameters of 24 h MII-pH monitoring and HRM [*M*(P*25*,*P75*)].

Group	NERD (n = 27)	RH (n = 30)	FH (n = 61)	*P* value
Acidic relux/times	39.0 (20.0, 59.0)	13.50 (4.8, 19.3)^a^	9.00 (4.5, 23.5)^a^	<0.001^* ^
Weakly acidic reflux/times	48.0 (30.0, 80.0)	37.0 (23.3, 60.3)	49.00 (31.5, 61.0)	0.186
Nonacidic reflux/times	7.00 (3.0, 17.0)	31.50 (9.8, 80.3)^a^	19.0 (8.5, 49.5)^a^	0.001^* ^
Upright AET/%	7.4 (5.6, 11.7)	0.8 (0.2, 2.2)^a^	0.8 (0.2, 2.0)^a^	<0.001^*^
Horizontal AET/%	6.0 (0.8, 9.4)	0 (0, 0.4)^a^	0 (0, 0.4)^a^	<0.001^* ^
Total AET/%	6.4 (4.8, 9.1)	0.5 (0.2, 1.9)^a^	0.7 (0.2, 1.5)^a^	<0.001^* ^
DeMeester score	22.8 (17.3, 34.0)	2.5 (0.9, 6.9)^a^	3.8 (1.1, 7.0)^a^	<0.001^* ^
Distal MNBI/Ω	1417 (1156, 1608)	2132 (1882, 2286)^a^	2524 (2341, 2958)^a,b^	<0.001^* ^
Proximal MNBI/Ω	2310 (1669, 2713)	2270 (1816, 2765)	2034 (1608, 2825)	0.640
PSPW index/%	9.1 (6.4, 12.3)	16.9 (12.7, 20.2)^a^	22.9 (16.7, 29.2)^a,b^	<0.001^* ^
Length of UES/cm	3.4 (3.2, 4.1)	3.5 (3.2, 4.1)	3.4 (3.1, 3.9)	0.674
Length of LES/cm	2.4 (2.2, 3.0)	2.5 (2.1, 2.8)	2.4 (2.1, 2.9)	0.858
ARP of UES/mmHg	28.0 (20.0, 71.0)	42.5 (19.8, 67.3)	33.0 (18.5, 50.0)	0.456
ARP of LES/mmHg	4.0 (2.0, 9.0)	8.5 (5.5, 15.3)^a^	11.0 (5.0, 16.0)^a^	0.010^* ^
RP of UES/mmHg	6.0 (1.0, 8.0)	4.5 (2.0, 7.3)	4.0 (2.0, 7.0)	0.439
RP of LES/mmHg	1.0 (0, 3.0)	3.0 (1.0, 5.3)^a^	3.0 (1.0, 5.0)^a^	0.034^* ^
IRP/mmHg	0.5 (−1.3, 2.7)	2.6 (0.9, 4.5)^a^	2.0 (1.1, 3.5)^a^	0.025^* ^
DCI/mmHg·s·cm	313.0 (109.0, 474.0)	309.0 (185.5, 761.3)	420.0 (221.0, 641.5)	0.140
DL/s	7.1 (6.5, 7.7)	7.1 (6.3, 8.3)	7.0 (6.2, 7.7)	0.561
FPL/cm	4.7 (2.4, 10.1)	4.5 (1.6, 8.3)	4.6 (1.8, 6.9)	0.436

Note: Compared with NERD group, ^a^*P *< 0.05; compared with RH group, ^b^*P* < 0.05. AET: acid exposure time; MNBI: mean nocturnal baseline impedance; PSPW: postreflux swallow-induced peristaltic wave; UES: upper esophageal sphincter; LES: lower esophagus sphincter; ARP: average resting pressure; RP: residual pressure; IRP: integrated relaxation pressure; DCI: distal contractile integral; DL: distal latency; FPL: fragmented peristalsis length.

### Parameters of 24 h MII-pH monitoring

The acid reflux time, AET percentage in the upright and lying positions, total AET percentage, and DeMeester score in NERD were greater than those in RH and FH (*P* < 0.001). The nonacid reflux, distal MNBI, and PSPW indices were lower than those in the RH and FH groups (*P*  < 0.001). The above reflux-related parameters were not significantly different between the RH group and the FH group (*P* > 0.05) (Table 2). However, the distal MNBI and PSPW of the RH group were significantly lower than those of the FH group (*P* < 0.05) and greater than those of the NERD group (*P* < 0.05) (Table 2).

**Fig 4:**
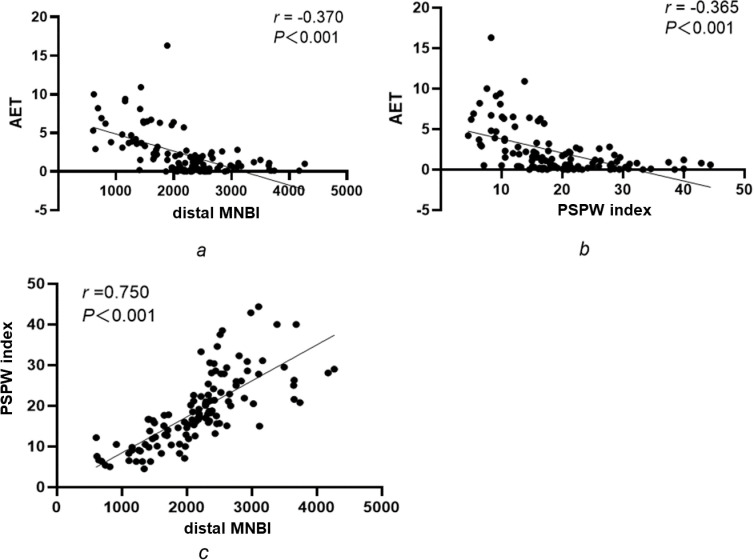
Correlations between AET, the distal MNBI and the PSPW index.

AET was negatively correlated with the distal MNBI (r =  −0.370, *P* < 0.001; Fig 4a) and PSPW index (r =  −0.365, *P* <  0.001; Fig 4b), whereas the PSPW index was positively correlated with the distal MNBI (r =  0.750, *P* <  0.001, *P* <  0.001, *P* <  0.001, *R* =  0.37, *P* <  0.001, *R* =  0.37, *P* <  0.001; Fig 4c).

**Fig 5:**
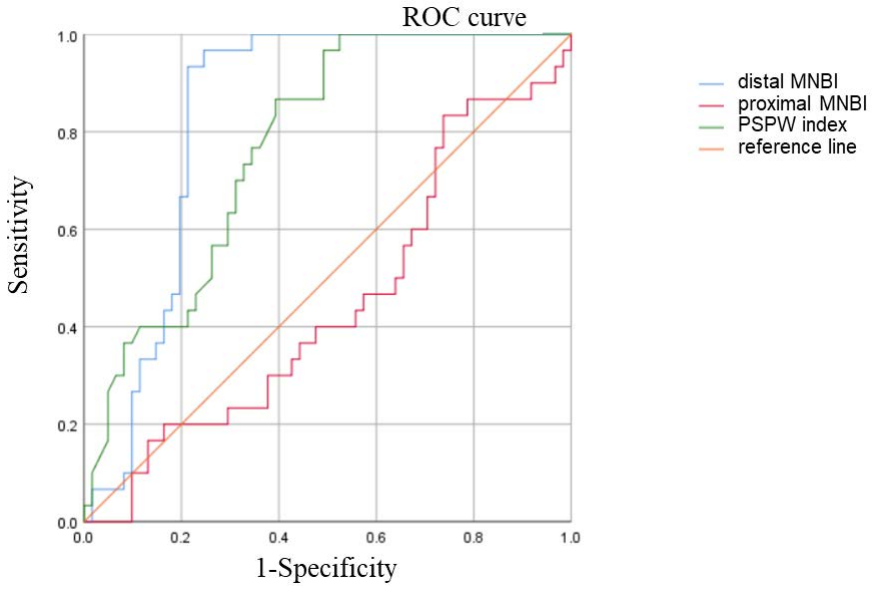
ROC curves of the proximal MNBI, distal MNBI, and PSPW index for the diagnosis of RH.

Additionally, we examined the diagnostic capability of novel parameters, including the proximal MNBI, distal MNBI, and PSPW indices, which have gained significant attention in recent years [9,14]. Our study assessed their effectiveness in distinguishing between FH and RH, with areas under the ROC curves of 0.448, 0.834, and 0.773, respectively. The Distal MNBI showed the best diagnostic value. The truncation value of the distal MNBI was 2346.2 Ω, the sensitivity was 96.7%, the specificity was 75.4%, and the YI value was 0.721 (Fig 5).

According to the truncation value of the distal MNBI obtained in this study (2346.2 Ω), 91 patients with negative endoscopy results and normal acid exposure were redivided into the RH group (44 patients (48%) and the FH group (47 patients (52%)). Among the 61 patients with FH diagnosed on the basis of SAP/SI, 15 (approximately 25%) were rediagnosed with RH. Among the 30 RH patients, 1 (3%) was rediagnosed with FH (Fig 1).

## Discussion

There were no statistically significant disparities among patients in the NERD, RH, and FH groups with respect to general information, suggesting that it is challenging to discriminate these three groups in clinical practice. Consequently, for endoscopically negative heartburn patients with persistent reflux symptoms, MII-pH monitoring is indispensable [10].

As far as our experimental results are concerned, RH patients are not as prone to comorbid FD-related symptoms as FH patients are, suggesting heterogeneity between RH patients and FGID patients [10]. In addition, the incidence of hiatal hernia was not significantly different between the RH and NERD groups. Moreover, the decreases in the distal MNBI and PSPW index suggest substantial pathological changes and esophageal clearance dysfunction in RH patients [10,25], which explains why antireflux surgery and PPI treatment are relatively more effective in RH patients than in FH patients.

According to our research, the disparities between RH and NERD are associated primarily with parameters that signify the severity of reflux, such as the quantity of acid reflux incidents, the proportion of AET, and the distal MNBI. We propose a hypothesis that RH and NERD are probably different phases of one illness; namely, RH might cause relatively milder damage than NERD. The esophageal antireflux mechanism gradually weakens in initially mild reflux, mucosal damage gradually intensifies, and RH eventually develops into NERD. Prospective trials on RH are needed to determine the process and prognosis of RH.

In addition, the distal MNBI has great diagnostic potential, and it is hoped that it can be included in the guideline at the earliest possible opportunity to assist with clinical diagnosis.

## Conclusions

There are distinct pathological alterations in RH, which markedly differ from those in other FGIDs, and the severity is less severe than that of NERD. Generally, RH has more similarities with NERD than with FH. It might be inappropriate to categorize RHs as FGIDs. To ascertain the prognosis of RH and the relationship between RH and NERD, prospective trials are needed.

## Supporting information

S1 File Original data of the study. (XLSX)
